# Case presentation of two patients using diagonal platform-switched double implants for maxillary single-first-molar replacement as the alternative of a single-tooth implant

**DOI:** 10.1186/s40729-015-0031-1

**Published:** 2015-11-12

**Authors:** Yasunori Hotta, Koji Ito, Shinichi Komatsu, Takashi Saito

**Affiliations:** Aichi Implant Center, Hotta Dental Clinic, 13, Morimaki-cho, Moriyama-ku Nagoya, 463-0073 Japan

**Keywords:** Implant, Double implants, Maxillary single-first-molar replacement, Diagonal implant placement, Platform-switched implant

## Abstract

A single-tooth implant restoration is generally performed for maxillary single-first-molar replacement. If the interdental space between the second premolar and the second molar is large enough, a double-implant placement can be performed to avoid creating mesiodistal cantilever and to distribute occlusal loading forces.

If there is not adequate space for a double-implant placement to be performed mesiodistally along the crest of the alveolar ridge line, they should be placed along a diagonal line offset lingually to increase the space. This procedure has two primary advantages. First, greater stability is provided by a double-implant placement. Resistance to lateral forces (palatal-buccal) is much stronger than two implants placed along the alveolar crest ridge line. Residual palatal and buccal bone can provide support against occlusal forces, provided that there is adequate residual bone in these regions.

If anatomical conditions are favorable, the placement of two diagonal implants in the palatal and buccal residual bones can be a rational procedure.

We report on two typical patients. The progress of these patients was followed using computed tomography for 7 and 6 years, respectively.

## Background

We report on two patients who were missing a maxillary first molar. In general clinical practice, only a single-tooth implant seems to be used as a prosthesis in patients with a missing maxillary molar tooth [1]. In addition, if there is insufficient residual bone between the bottom of the sinus and the crest of the alveolar ridge, GBR (guided bone regeneration) technique [2] or sinus elevation [3] surgery is required. Even though these procedures can promote osteogenesis, whether the quality of newly formed bone is similar to that of the existing residual bone remains unclear [4–8]. If there is enough residual D2 or D3 bone (Misch Bone Density Classification) [9] for implant placement, the use of such bone is recommended whenever possible. If we accept the above, a double-implant placement [10, 11] inserted in the buccal and palatal sides, not the center of the alveolar ridge, may be a reasonable option for maxillary-first-molar replacement because these regions sometimes retain more bone volume than the center of defects.

## Case presentation

### Patient 1

**Fig. 1:**
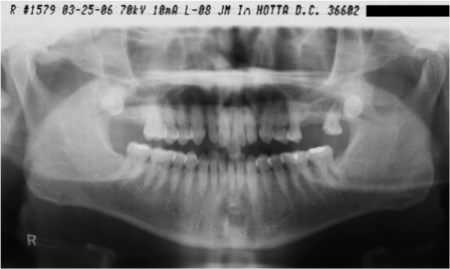
Case 1. March 2006. A panoramic X-ray film obtained before treatment

**Fig. 2:**
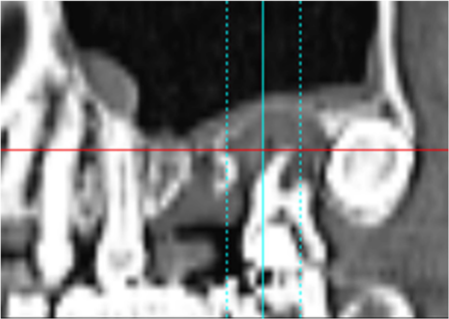
Case 1. May 2006. Preoperative panoramic slice of MDCT scan. There was severe bone resorption in the region of #26 and lesions around the root apexes of #27

The patient was a 51-year-old man who was a nonsmoker. He was referred to my clinic in April 2004. The #26 tooth had already been extracted because of severe periodontitis. He returned to my clinic in March 2006 to receive implant treatment (Fig. 1). The medical history was unremarkable. A hematological analysis was performed to assess his overall medical status. The #26 tooth was confirmed to be missing, and there was severe bone resorption around the buccal roots of #27, associated with an apical lesion (Fig. 2).

**Fig. 3:**
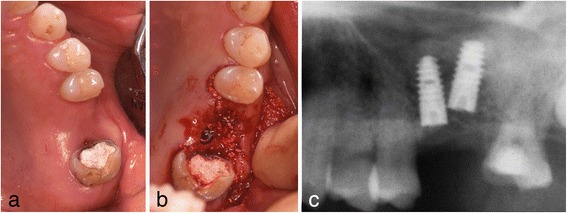
Case 1. May 2006. **a** Before implant surgery. **b** Double implants were placed diagonally in the missing area. **c** Magnified panoramic X-ray film obtained immediately after insertion of double implants

**Fig. 4:**
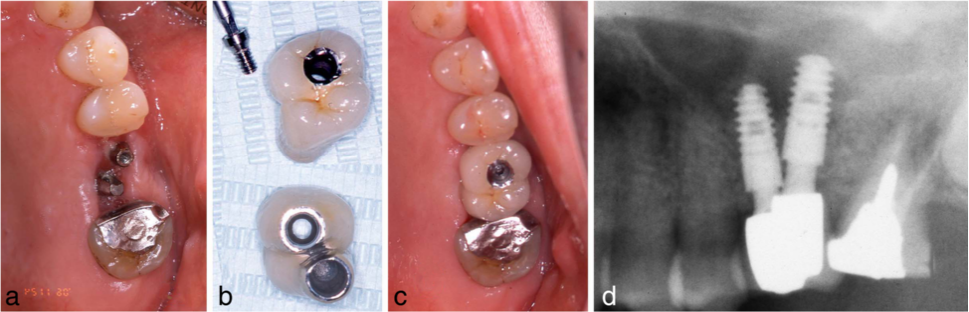
Case 1. November 2006. Six months after implant insertion. **a**–**c** The superstructure was attached with a screw. **d** Magnified panoramic X-ray film obtained immediately after superstructure placement

In May of the same year, double implants (Ankylos implants [12]; diameter, 3.5 mm; length, 9.5 mm; DENTSPLY, made in Germany) were placed diagonally in the defect with sinus elevation (crestal approach) (Fig. 3). In addition, Emdogain (enamel matrix protein) [13–15] was applied to the root surface of #27. Then, endodontic treatment of #27 was performed (Fig. 4d). Six months after implant surgery, the screw-retained superstructure was seated (Fig. 4).

### Patient 2

Similar to the first patient, our second patient was a nonsmoking man, 43 years of age. He was referred to my clinic by another dentist in January 2008 and was first examined at that time. He complained of pain due to serious periodontitis in #26. The medical history was unremarkable. He was examined in a similar manner as patient 1.

**Fig. 5:**
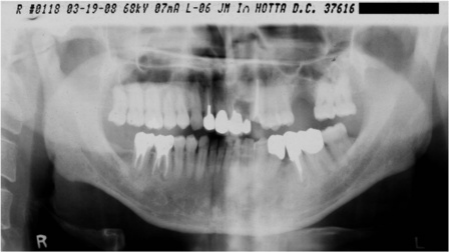
Case 2. March 2008. Panoramic X-ray film taken before #26 GBR

**Fig. 6:**
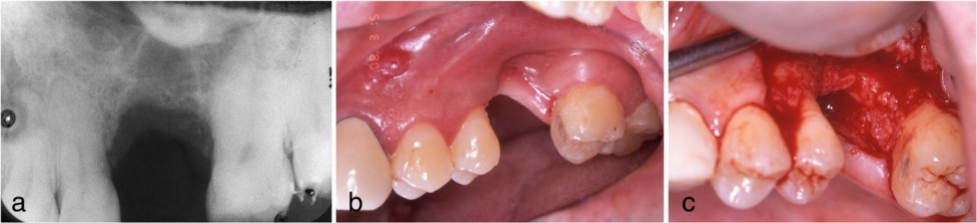
Case 2. March 2008. Two months after extraction of #26. **a** Periapical X-ray film of #26 before surgery. **b**, **c** Oral view shows very severe bone resorption, especially vertically

**Fig. 7:**
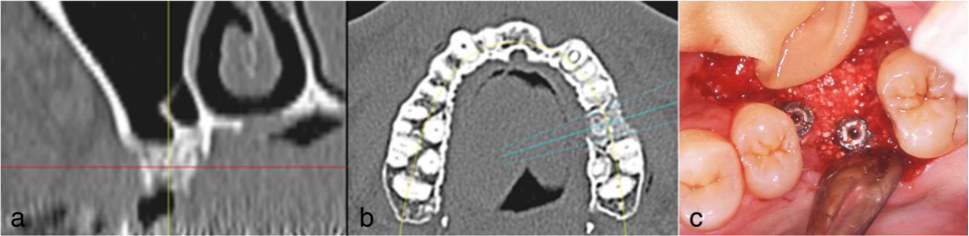
Case 2. June 2008. **a**, **b** 3 months after GBR without implant insertion, MDCT was performed to evaluate. **c** Double implants were placed diagonally

**Fig. 8:**
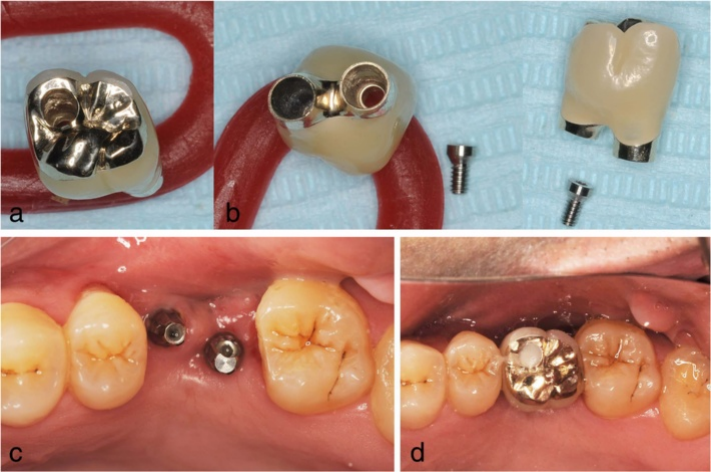
Case 2. November 2008. Five months after implant insertion. **a** A fitted superstructure was made. **b** Before the placement of the superstructure. The double implants were placed diagonally. **c** The superstructure was attached with a screw as in case 1.**d** After placement of superstructure

In the same month, #26 was extracted. After extraction, bone resorption including the buccal cortical plate occurred more severely than in most patients (Figs. 5 and 6). Two months after extraction, only GBR was performed to repair the bone defect. Double implants (Ankylos implants; diameter, 3.5 mm; length, 9.5 and 11 mm) were placed diagonally in the missing region 3 months after GBR (Fig. 7). In November of the same year, a screw-retained superstructure was seated (Fig. 8).

### Treatment results

**Fig. 9:**
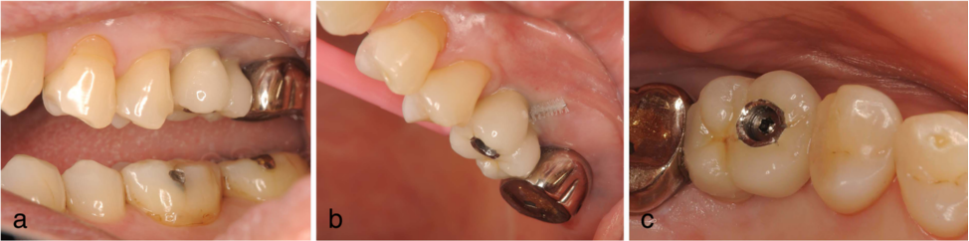
Case 1. April 2013. Six years and 5 months after superstructure placement. **a** The buccal oral view of the superstructure. **b** An interdental brush can easily clean the interimplant space. **c** April 2014, 7 years and 5 months after superstructure placement

**Fig. 10:**
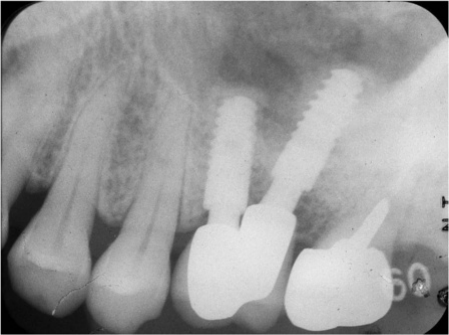
Case 1. Periapical X-ray film, April 2014. Seven years and 5 months after superstructure placement, showing no bone resorption around the double implant necks

**Fig. 11:**
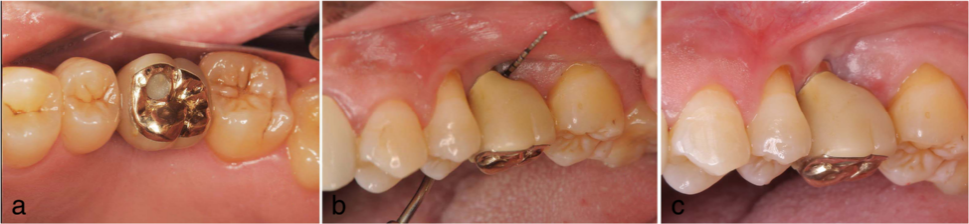
Case 2. **a** April 2013. Four years and 5 months after placement of the superstructure. Occlusal view. **b** Periodontal probe can easily pass through the interimplant space, allowing for easy cleaning and maintenance. **c** June 2015, 6 years and 7 months after placement of the superstructure

**Fig. 12:**
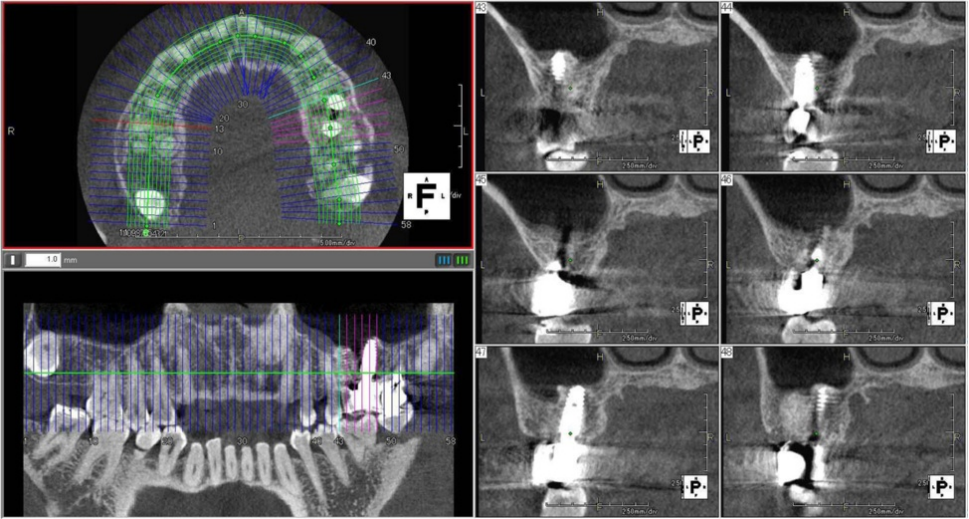
Case 1. April 2013. Cone-beam CT obtained 6 years and 5 months after superstructure placement. The double implants were surrounded by an adequate amount of hard tissue that acted as bone

**Fig. 13:**
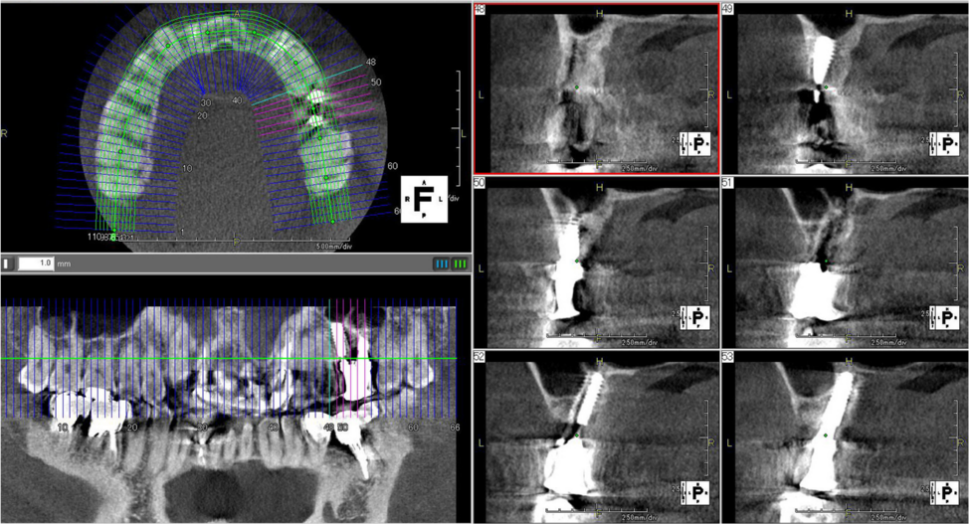
Case 2. Cone-beam CT scan obtained in February 2013 (4 years and 3 months after superstructure placement), showing that the double implants were surrounded by enough hard tissue to support the double implants

The patients’ clinical progress has been good as of 7 years and 5 months in patient 1 (Figs. 9 and 10) and 6 years and 7 months in patient 2 (Fig. 11). Computed tomographic (CT) scans showed that both patients’ implants were inserted at appropriate positions, as planned before surgery (Figs. 12 and 13).

### Discussion

Insertion of a single implant for single molar replacement is established practice. Levin et al. reported that a single implant can serve as a good long-term and predictable treatment modality to replace a single molar with low complication and failure rates [16].

However, weak points of single-first-molar replacement were reported.

Mazor et al. reported that 33 patients received 66 narrow double implants of 3 mm replacing 33 missing first molars. All implants survived the follow-up time of 10 to 18 months. Wide-diameter implants are a suitable alternative for replacing a missing molar in some cases, but there has been a greater incidence of screw loosening, resulting in a higher failure rate. However, the use of a double-implant placement has been successfully demonstrated to be a functional and more biomechanically sound method of molar replacement. He concluded that replacing a single missing molar with two narrow dental implants might serve as a viable treatment option providing good and predictable long-term results [17].

Sullivan reported that when using standard-diameter 3.75-mm titanium screw implants in molar regions, up to 14 % of single-tooth, molar implants could fracture, so there remains an unacceptable risk of implant fracture [18].

In addition, a double-implant placement has been reported to be superior to a single stand implant. The advantages and disadvantages of a single standard implant, a wide-diameter implant, and double implants for single-molar replacement have been debated with biomechanics [10, 11, 19–21].

If there is 13 mm between the maxillary second premolar and the second molar, a double-implant placement can be performed in the crest of the alveolar ridge line. When 3.5-mm diameter implants are used, there should be a minimum space of 3 mm between implants [22] and 1.5 mm between the implants and adjacent teeth [23] (1.5 + 3.5 + 3 + 3.5 + 1.5 = 13 mm). However, the average Japanese mesiodistal dimension of the maxillary first molar is between 10 and 11 mm [24]. Therefore, if a double-implant placement is used, it has to be placed diagonally to create a larger inter-implant space and accommodate international implant sizing [1].

As for the vertical bone dimension, if there is insufficient residual bone volume between the bottom of the sinus and the crest of the alveolar ridge, GBR or sinus elevation surgery is required for all implants. If there is sufficient existing residual D2 or D3 bone near the buccal and palatal cortical plates, the use of such bone is recommended whenever possible. If double implants can engage these cortical plates, greater support can be obtained with diagonal double implants than with a single stand implant.

In both patients, we used Ankylos implants, which have a medialized step between the implant body and abutment. Therefore, Ankylos is a platform-switched implant. The benefits of this shape have been reported. With platform-switched implants, the shape affects the cortical bone more than the trabecular bone [25], which contributes to the maintenance of inter-implant bone height and soft tissue level [26] and reduces bone loss [27]. The horizontal discrepancy between the outer edge of the implant platform and the implant-abutment interface influences post-restorative biologic processes [28]. This horizontal discrepancy may reduce bone resorption around the implant neck because it horizontally offsets the bone away from the microgap that exists between the implant and abutment. This increased distance is thought to attenuate the loss of crestal bone height [29].

Effects of the inter-implant distance between platform-switched implants have been reported. Even when the inter-implant distance was less than 3 mm, crestal bone resorption was prevented [30-34]. Previous studies thus support the advantages of platform-switched implants as compared with platform-matched implants when double implants are placed adjacently.

In patient 1, the platform-switched double-implant placement was performed very close to each other (Fig. 3). If platform-matched implants had been used, inter-implant bone loss might have occurred. Fortunately, there was no evidence of bone resorption on the periapical X-ray obtained 7 years and 5 months after superstructure placement (Fig. 10). In addition, there has been no problem with the emergence profile because platform-switched implants provide a larger volume of soft tissue around the connecting area between the implant and abutment.

In patient 2, severe bone resorption including the buccal cortical plate occurred after #26 extraction and was greater than we had anticipated. Therefore, only GBR was performed without implant insertion to repair the severe bone resorption (Fig. 6). CT was performed to evaluate the GBR site after 3 months. Then, a double-implant placement was performed diagonally with another session of GBR to increase bone volume (Fig. 7).

**Fig. 14:**
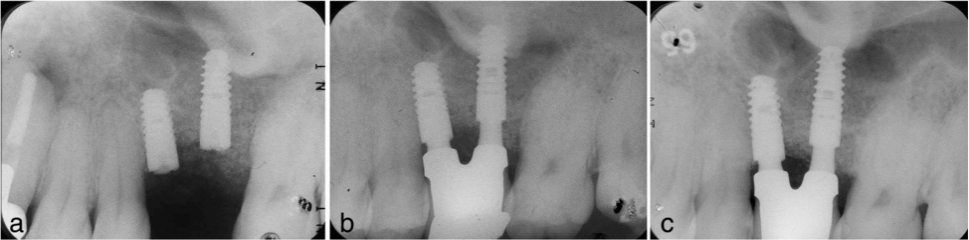
Case 2. Periapical X-ray films showing progression of intraimplant bone formation from implant date to 3 years and 8 months after procedure. **a** June 2008. **b** November 2008. **c** February 2012

The use of platform-switched implants has been associated with coronal extension of the interimplant bone peak beyond an imaginary line connecting the two implant-abutment interfaces [22]. In patient 2, even though the double-implant placement was performed adjacently, new bone formation over the implant shoulder was fortunately confirmed on a periapical X-ray (Fig. 14).

In diagonal double-implant placement, the mesial implant should be placed more buccally and the distal implant more palatally to improve esthetics [1]. In addition, oral hygiene is a very important factor related to inter-implant space. It might be easier for a patient to use an interdental brush from the palatal side rather than from the buccal side, especially in the maxillary molar region.

On the other hand, Bhat and Handelsman reported that there is a surgical risk due to the precision needed in a double-implant placement, because the mesiodistal space is often very limited. Therefore, any misplacement of double implants will ruin the mesial or the distal site, and misplacement also may damage the adjacent roots or compromise the embrasure space, so that only one implant can be placed [35]. This kind of careful attention should be paid when using a double-implant placement in single molar missing.

## Conclusions

We described two patients in whom diagonal platform-switched double-implant placement was performed for maxillary single-first-molar replacement. In such patients, diagonal double implants have some advantages over wide-diameter implants. There was no bone resorption around double implants’ necks. We therefore consider this method as one option for maxillary single-first-molar replacement. 

## Consent

Written informed consent was obtained from the patient for publication of this case report and any accompanying images. A copy of the written consent is available for review by the Editor-in-Chief of this journal.
